# INCOME INEQUALITY, INCOME, AND HEALTH OVER THE LATER LIFE COURSE IN EUROPE: A LONGITUDINAL AND CROSS-NATIONAL STUDY

**DOI:** 10.1093/geroni/igae098.3211

**Published:** 2024-12-31

**Authors:** Mengling Cheng, Nicolas Sommet, Dario Spini

**Affiliations:** University of Lausanne / East China University of Science and Technology, Switzerland; University of Lausanne, Lausanne, Vaud, Switzerland; University of Lausanne, Lausanne, Vaud, Switzerland

## Abstract

Evidence from the U.S., UK, and Europe documented that income inequality has modest effects on the social gradient of health. However, few studies investigate how the effect of income inequality on the social gradient of health evolves over the life course. This study aims to examine to what extent, and in what ways, does the effect of income on later-life health trajectory vary across more unequal and less unequal societies. This study used data from the Standardized World Income Inequality Database (SWIID, 2004-2020) and the Survey of Health, Ageing and Retirement in Europe (SHARE, 2004-2010). We used national-level Gini index to measure income inequality. We used life satisfaction as the health outcome. We performed multilevel modelling to analyze the data in which observations (level 1) are nested in participants (level 2), country-years (level 3), and countries (level 4). Our analysis revealed three findings. First, the effect of income on life satisfaction among older adults is more pronounced in unequal countries. Second, the effect of income inequality on life satisfaction weakens as individuals age. Third, the importance of income on life satisfaction across societies of varying levels of income inequality decreases over the life course of an individual. Findings from this study show that contextual-level income inequality can modify the effects of individual income on health as individuals age. This can help the policy makers and practitioners to target the vulnerable population and offer more effective intervention.

